# Highly divergent dengue virus type 1 genotype sets a new distance record

**DOI:** 10.1038/srep22356

**Published:** 2016-02-29

**Authors:** Alyssa T. Pyke, Peter R. Moore, Carmel T. Taylor, Sonja Hall-Mendelin, Jane N. Cameron, Glen R. Hewitson, Dennis S. Pukallus, Bixing Huang, David Warrilow, Andrew F. van den Hurk

**Affiliations:** 1 Public Health Virology Laboratory, Forensic and Scientific Services, Coopers Plains, Queensland, Australia

## Abstract

Dengue viruses (DENVs) are the leading cause of mosquito-borne viral disease of humans. They exist in both endemic and sylvatic ecotypes. In 2014, a viremic patient who had recently visited the rainforests of Brunei returned to Australia displaying symptoms consistent with DENV infection. A unique DENV strain was subsequently isolated from the patient, which we propose belongs to a new genotype within DENV serotype 1 (DENV-1). Bayesian evolutionary phylogenetic analysis suggests that the putative sylvatic DENV-1 Brunei 2014 (Brun2014) is the most divergent DENV-1 yet recorded and increases the time to the most recent common ancestor (MRCA) for DENV-1 from ≈120 years to ≈315 years. DENV-1 classification of the Brun2014 strain was further supported by monoclonal antibody serotyping data. Phenotypic characterization demonstrated that Brun2014 replication rates in mosquito cells and infection rates in *Aedes aegypti* mosquitoes were not significantly different from an epidemic DENV-1 strain. Given its ability to cause human illness and infect *Ae. aegypti*, potential urban spillover and clinical disease from further Brun2014 transmission cannot be discounted.

Dengue is the most prevalent mosquito-borne viral disease in tropical and subtropical countries, causing approximately 390 million infections annually^1^. Dengue viruses (DENVs) belong to the genus *Flavivirus,* family *Flaviviridae* and have been antigenically classified into one of four individual serotypes (DENV 1–4)^2^. Phylogenetic studies have provided strong evidence that each DENV serotype evolved from ancestral sylvatic progenitors in an independent manner. As a consequence of evolutionary processes and incidental spillover events, sylvatic DENV has emerged in urban cycles between peridomestic *Aedes aegypti* or *Aedes albopictus* mosquitoes and human amplifying hosts^3^,^4^,^5^,^6^.

Sylvatic DENV transmission between non-human primates and arboreal *Aedes* mosquitoes occurs in West Africa and the Malaysian peninsula, where it continues to cause sporadic human cases^4^,^7^,^8^,^9^,^10^. However, the frequency of spillover events and human infection rates remains largely unknown and is exacerbated by the inherent difficulty in clinically distinguishing between sylvatic and endemic/epidemic human DENV infections^7^,^8^,^9^,^10^,^11^,^12^. Furthermore, there is ongoing contention surrounding the pathological and epidemic potential of sylvatic DENV, risks posed to public health and implications for future vaccines^6^,^12^,^13^.

In the current report, we describe the genotypic and phenotypic characterization of a previously unknown and highly divergent DENV-1 strain from Brunei which was imported into Australia by a viremic patient. To date, phylogenetic classification of DENV-1 has identified five distinct genotypes (I–V) including genotype III in which the proposed Malaysian sylvatic 1972 (monkey) and 2005 (human) strains are represented^3^,^6^,^10^,^14^,^15^. Here, we provide novel phylogenetic evidence that the DENV-1 Brunei 2014 strain (Brun2014) represents a new and highly divergent genotype (designated genotype VI). It has been suggested previously that sylvatic DENV-1 diverged from human strains between ≈100 years and ≈200 years ago in Asia before its introduction into Africa, the Americas and the Pacific^15^,^16^. We demonstrate a significantly revised estimate of the time to the most recent common ancestor (MRCA) for DENV-1 which unambiguously places Brun2014 in a basal position within phylogenetic trees relative to any previously reported sylvatic or endemic/epidemic DENV-1 sequences. Importantly, the isolation of the Brun2014 strain and reporting of related findings underscores the genuine threat posed by sylvatic DENV spillover and potential consequences to human health. This discovery enhances the current understanding of DENV evolutionary diversity and dynamics and will enable new insights into sylvatic strain transmission and pathogenicity.

## Results

### Sequencing

**Figure 1:**
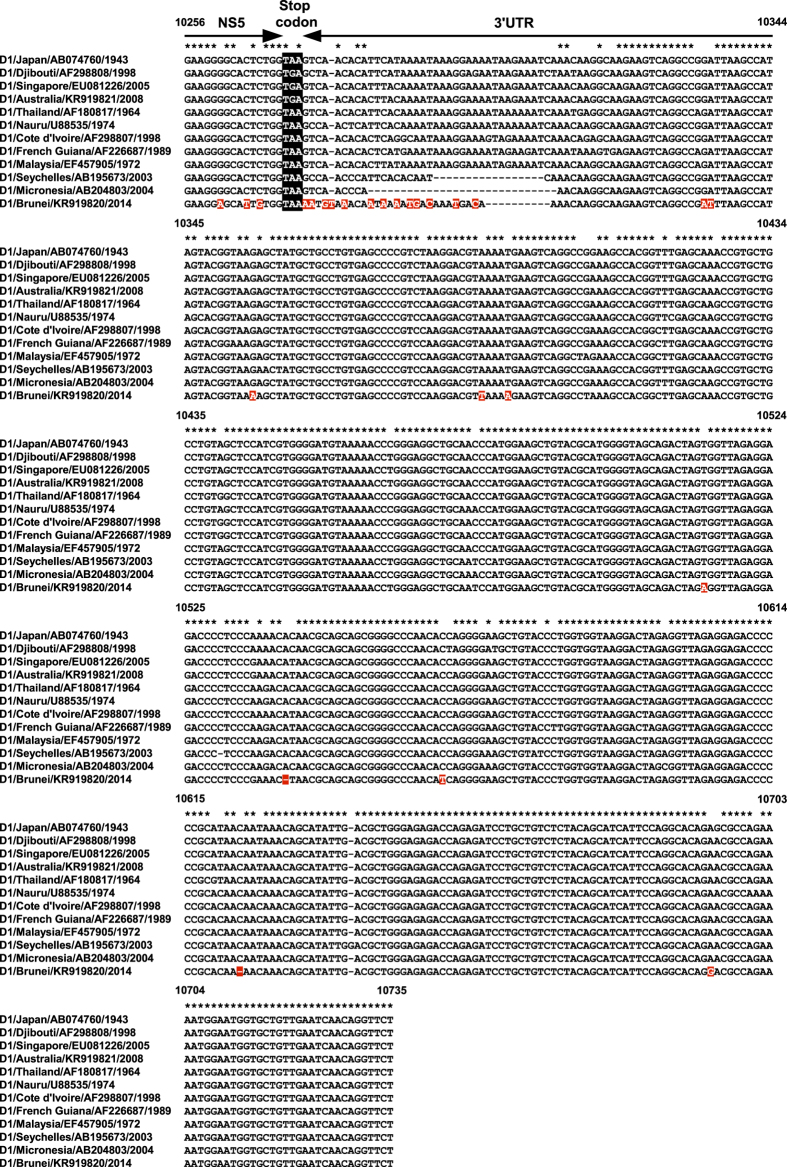
Alignment of nucleotide consensus sequences of the dengue virus serotype 1 (DENV-1) 3′ untranslated region (UTR). Nucleotide position numbers correspond to the Malaysian 1972 strain, P72-1244 (GenBank accession number EF457905). Nucleotide identity is indicated by stars (*) and deletions are shown by dashes (−). Nucleotide changes unique to Brun2014 are shaded in red.

Whole genome sequences of Brun2014 and an outbreak DENV-1 strain (TSV08) isolated from a patient from Townsville, north Queensland in 2008 were determined and deposited on GenBank (accession numbers KR919820 and KR919821 respectively). A complete list of DENV isolates used in the study and their GenBank accession numbers is provided in Supplementary Table S1. The Brun2014 sequence contained a large number of nucleotide differences compared to other available DENV sequences and was found to have only 83% nucleotide identity to the most closely related DENV-1 strains, with highest similarity to the sylvatic 1972 Malaysian isolate, P72-1244 (GenBank accession number EF457905). However, both the 5′ and 3′ untranslated regions (UTRs) of Brun2014 were more conserved than the coding sequence when compared with other DENV-1 viruses and reached maximal nucleotide identities of 100% and 96% respectively. The Brun2014 3′UTR sequence demonstrated variability at multiple sites and was found to be 11 nucleotides shorter in length (451 nt) than most representative DENV-1 3′UTR sequences (462 nt) including isolates P72-1244 and TSV08. The high degree of Brun2014 sequence heterogeneity was most notable in the hypervariable region immediately downstream of the non-structural protein 5 (NS5)^17^ and this complicated the sequence alignment (Fig. 1). Indeed, 9 of the 11 nucleotide deletions in the Brun2014 3′UTR were observed in this region. Whilst the remaining downstream 3′UTR sequence was found to be highly conserved to other DENV-1 sequences, two single nucleotide deletions were observed for the Brun2014 sequence corresponding to P72-1244 (GenBank accession number EF457905) genome positions 10540 and 10623 respectively.

Similarly, comparison of the Brun2014 coding region amino acid sequences with other available DENV sequences revealed highest amino acid identity (94%) with P72-1244. A large number of nucleotide differences within the Brun2014 coding region sequence were nonsynonymous, many of which resulted in amino acid changes that were unique to Brun2014 (Supplementary Table S2). A summary of the complete coding region nucleotide and amino acid pairwise differences between Brun2014 and 53 other DENV-1 sequences is summarised in Supplementary Fig. S1. Average differences across the whole genome were estimated to be 17.9% (minimum 17.4%, maximum 18.3%) and 6.8% (minimum 6.0%, maximum 7.9%) in nucleotide and amino acid sequences respectively.

### Evolutionary analysis and phylogenetics

**Figure 2:**
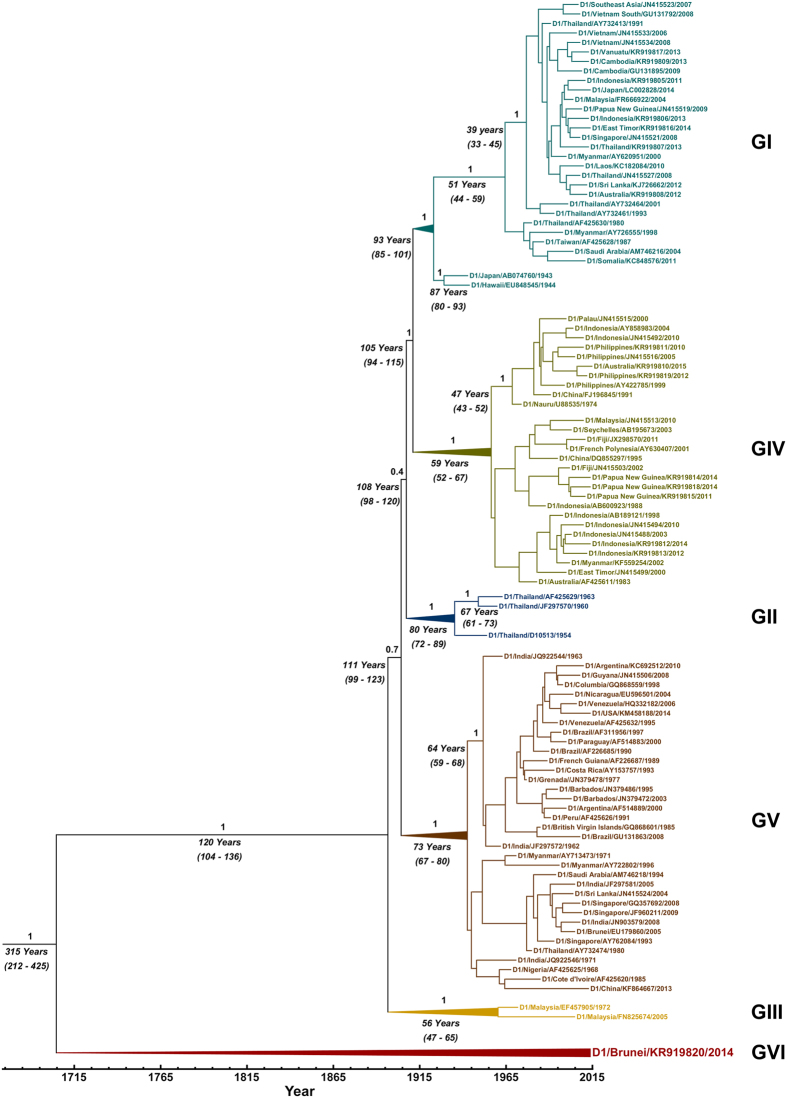
Maximum clade credibility tree of 100 dengue virus serotype 1 (DENV-1) E gene sequences. All five major DENV-1 genotypes (I to V) are shown and compared to a new postulated sylvatic genotype (VI) containing the previously unknown strain, Brun2014. Horizontal branch lengths are drawn to scale and are proportional to time. Posterior probability values (1.0) are shown for key nodes together with corresponding divergence times and respective 95% high probability densities (in parentheses).

**Figure 3:**
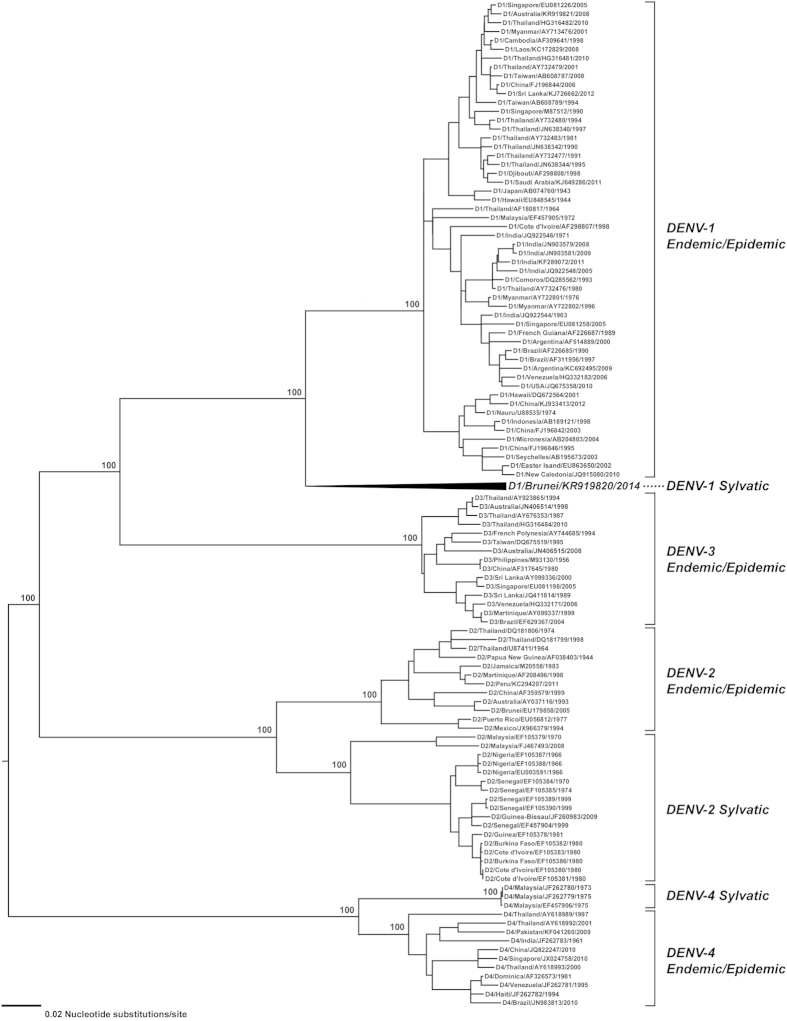
Maximum likelihood phylogenetic tree of whole genome coding regions of 113 dengue virus (DENV) strains representing all four serotypes. The DENV 1–4 serotypes are shown as either human endemic/epidemic or sylvatic lineages with the exception of DENV-3 for which sylvatic sequences are not available. Zika virus (Uganda 1947, GenBank accession number AY632535) was used as an outgroup. Horizontal branch lengths are proportional to the bar representing the number of nucleotide substitutions/site. Percentage bootstrap support values determined from 1,000 replicates are shown for key nodes.

For each of the coding sequence (n = 54) and E gene (n = 100) DENV-1 datasets studied, the estimated mean rate of nucleotide substitution was 6.47 × 10^−4^ and 7.08 × 10^−4^ substitutions/site/year respectively. Sampled from a broad geographical area and incorporating all available DENV-1 sylvatic sequences, a phylogenetic tree was constructed from 100 DENV-1 E gene sequences (Fig. 2). Notably, the Brun2014 strain was shown to form a distinctly new DENV-1 genotype^3^ which we propose is genotype VI. This finding, and the basal positioning of Brun2014 relative to the five existing DENV-1 genotypes, is further supported by the phylogenetic trees constructed from coding sequences of DENV-1 (Supplementary Fig. S2) and DENV 1–4 serotypes (Fig. 3).

The time to the MRCA for DENV-1 was ≈315 years with 95% high probability densities (HPD) between 212 and 425 years (Fig. 2). Similar values were obtained with the phylogenetic tree constructed from the complete coding sequences of 54 DENV-1 sequences (Supplementary Fig. S2).

### Selection pressure analysis

Selection pressure analysis was performed on the E gene (n = 100) and complete coding regions (n = 54) of the studied DENV-1 sequences (Supplementary Table S1). Resulting ω ratios were low (0.060–0.081) indicating codons were primarily under negative selection. Analysis performed separately on the E gene sequences of the three postulated DENV-1 sylvatic strains namely Brun2014, P72-1244 and the Malaysian 2005 strain 36046 (GenBank accession number FN825674) also demonstrated very low ω ratios (0.026–0.033) indicating the absence of positive selection. These findings are consistent with data from previous DENV-1 studies and indeed reflects the overall consensus that negative selection is the major selecting pressure influencing DENV 1–4 genomes^5^,^18^,^19^,^20^,^21^.

### Kinetic replication curve experiments

**Figure 4:**
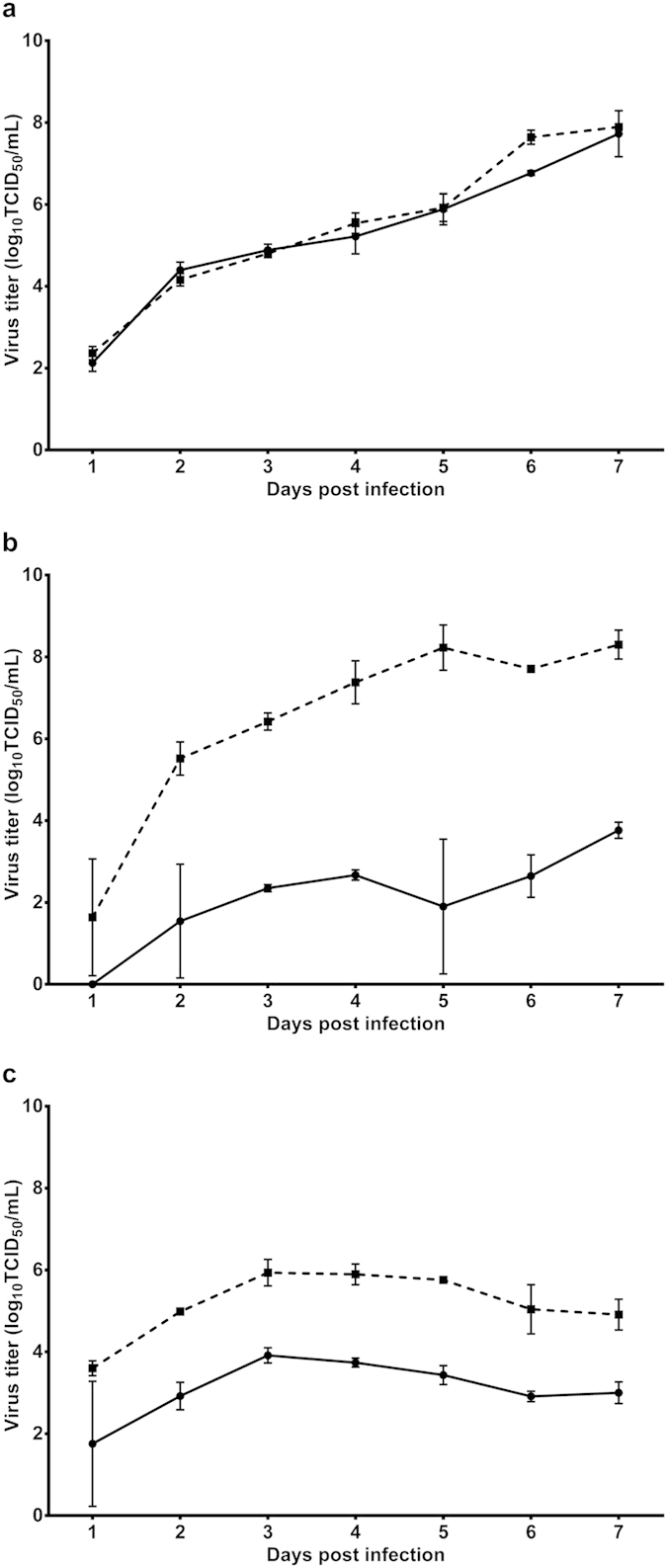
Kinetic replication curve analyses. Comparison of replication curves for Brun2014 (solid line) and the urban epidemic strain TSV08 (dashed line) are shown for C6/36 mosquito cells (**a**) and mammalian Vero and Huh-7 (**b,c** respectively). Viruses were infected at a multiplicity of infection (m.o.i.) of 0.01 based on respective titers obtained in C6/36 cells. Mean (±SD) viral titers resulting from the average of independent replicates are plotted for each virus. Virus samples were collected daily for 7 days.

Relative replication rate analyses were performed to compare Brun2014 replication in C6/36 mosquito, Vero and Huh-7 mammalian cells with the urban outbreak TSV08 DENV-1 strain (Fig. 4). Although cultured C6/36 and Vero cells deficient in RNAi^22^ and interferon pathways^23^ respectively are considered imperfect models for DENV infectivity, they can be useful in demonstrating initial phenotypic variation between viruses^24^. In all cell lines, 2-way repeated measures ANOVA revealed that day post infection significantly (*P* < 0.001) affected mean virus titers. Viral replication of Brun2014 and TSV08 continued to increase in both C6/36 and Vero cells until maximum titers were obtained on day 7, whereas in Huh-7 cells, both viruses produced peak titers on day 3, after which titers plateaued and subsequently decreased, possibly due to pronounced cytopathic effect (CPE). In C6/36 cells, no significant difference in mean viral titers was observed between the two viruses (F_1,2_ = 15.6, *P* = 0.059, 2-way repeated measures ANOVA) and there was no significant interaction between virus strain and day post infection (F_6,12_ = 2.3, *P*  = 0.105). Contrastingly, in Vero cells, virus strain (F_1,2_ = 361.8, *P*  = 0.003) significantly affected mean titers, and there was a significant interaction with day post infection (F_6,12_ = 4.356, *P*  = 0.015). A similar result was obtained with Huh-7 cells, in that virus strain (F_1,2_ = 456.0, *P*  = 0.002) significantly affected mean titers, but without any significant interaction with day post infection (F_6,12_ = 0.155, *P*  = 0.984). Collectively, these findings suggest that Brun2014 varied significantly in its ability to replicate in mammalian cells compared with TSV08. This may indicate an altered host cell tropism which could have been influenced by factors such as reduced cellular adaptation or temperature sensitivity of Brun2014.

### Vector competence experiments

We undertook vector competence experiments to determine whether Brun2014 could infect and disseminate in *Ae. aegypti*, the primary epidemic vector in urban areas throughout the world, including north Queensland^25^,^26^. We also assessed *Ae. notoscriptus,* a member of the subgenus *Finlaya* in the genus *Aedes*, as it is in the same subgenus as *Ae. niveus* complex mosquitoes, which are considered to be the vectors of sylvatic DENV in Malaysia^12^. Of note, *Ae. notoscriptus* is also the dominant container-inhabiting *Aedes* mosquito in the city of Brisbane where the Brun2014 patient resided^27^. Infection and dissemination rates were compared to the TSV08 outbreak strain. Although a salivary gland infection barrier cannot be discounted, disseminated infection rates (percentage of mosquitoes with virus in their legs and wings) are indicative of the vector competence of mosquitoes for DENV^28^,^29^.

Chi squared tests for heterogeneity between studies showed no significant difference in infection or dissemination rates for either species or virus strain across studies, so data for each mosquito species and virus strain was pooled for analysis. Although infection and dissemination rates were lower in *Ae. aegypti* and *Ae. notoscriptus* exposed to Brun2014 compared to those exposed to TSV08, the difference was not significant (*P* > 0.05) (Table 1). For both viruses, the infection rates in *Ae. notoscriptus* were not significantly different (*P* > 0.05) from those observed for *Ae. aegypti*. However, dissemination rates were lower in *Ae. notoscriptus* compared to *Ae. aegypti* for both viruses, with the difference being significant (*P* < 0.05) in mosquitoes exposed to TSV08. *Ae. aegypti* does not appear to express a midgut escape barrier, as once *Ae. aegypti* was infected with Brun2014 and TSV08, 87.5% and 90% of mosquitoes, respectively, developed a disseminated infection. In contrast, 16.7% and 25.0% of *Ae. notoscriptus* infected with Brun2014 and TSV08, respectively, developed a disseminated infection, suggesting that a midgut escape barrier limits the dissemination of the virus from infected midguts in this species.

### Immunological characterization of Brun2014

**Figure 5:**
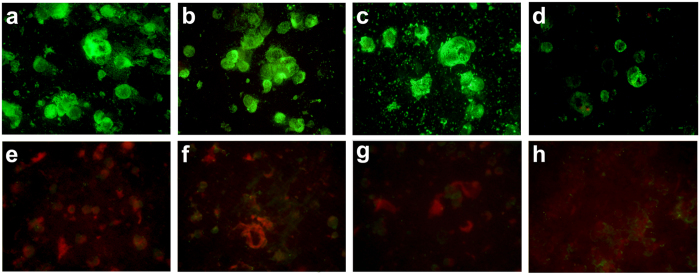
Immunofluorescent antibody assay of Brun2014 infected C6/36 *Ae. albopictus* cells. Cells were stained day 5 post infection using anti-flavivirus monoclonal antibody (**a**) 4G2 and the following anti-DENV monoclonal antibodies, namely (**b**) DENV 1-4 complex HB114, (**c**) DENV-1 HB47, (**d**) DENV-1 M17, (**e**) DENV-2 HB46, (**f**) DENV-3 11D5 and (**g**) DENV-4 HB48. An uninfected C6/36 cell control is shown (**h**) which was stained using the DENV-1 HB47 monoclonal antibody. Positive reactivity is indicated by green fluorescence. Microscope magnification ×400.

The Brun2014 strain was characterized immunologically using a panel of mouse monoclonal antibodies. Immunofluorescent antibody (IFA) staining of Brun2014 infected C6/36 cells using a panel of flavivirus-specific (4G2) and DENV 1–4 monoclonal antibodies is shown in Fig. 5. The Brun2014 strain reacted strongly with 4G2, DENV 1–4 complex HB114 and DENV-1 HB47 antibodies and was weakly reactive with DENV-1 M17 antibody. No reactivity was detected using DENV 2–4 monoclonal antibodies indicating the Brun2014 virus belonged to the DENV-1 serotype.

## Discussion

In the current study, we have described the discovery of a new DENV-1 genotype VI which to our knowledge represents the most divergent sylvatic strain recorded for this serotype. The revised estimate of the time to the MRCA for DENV-1 was ≈300 years as shown by both E gene and complete coding region phylogenetics, which is ≈150 years earlier than prior estimates^16^.

The mean rate of nucleotide substitution for analysed DENV-1 sequences was between 6.47 × 10^−4^ and 7.08 × 10^−4^ substitutions/site/year and similar to previously reported rates^15^. The distinctly divergent Brun2014 sequence was most similar to the 1972 Malaysian sylvatic P72-1244 strain further supporting the hypothesis that DENV-1 evolved initially in Asia before later dispersal to Africa and the Americas^15^. Interestingly, it has been suggested that P72-1244 may in fact represent a spillback strain from humans which was found circulating in the Malaysian forest^5^,^12^. This has been supported previously by E gene phylogenetics and the finding that the 2005 human DENV-1 Malaysian strain 36046/05 (GenBank accession number FN825674) clusters with P72-1244^10^,^12^. In the current study, we have demonstrated similar findings (Fig. 2) and whole genome maximum likelihood phylogenetics (Fig. 3) places P72-1244 with other DENV-1 endemic/epidemic strains. Further, our phylogenetic analyses show that Brun2014 is unambiguously placed in genotype VI which is situated in a basal position relative to P72-1244 and all other previously reported DENV-1 strains. This strongly supports the hypothesis that in contrast to Brun2014, P72-1244 represents a spillback strain from human endemic/epidemic transmission cycles and therefore is not a genuine sylvatic DENV-1 strain.

Average nucleotide and amino acid differences between Brun2014 and other DENV-1 strains across the coding region were high (17.9% and 6.8% respectively) further supporting a new genotype designation^3^. Similar findings were obtained for the surface E protein which is important for DENV cell entry^6^ and a major immune target for DENV vaccines, including the most currently advanced DENV vaccine candidate, CYD-TDV^30^,^31^. Of note, no amino acid mutations were observed in the Brun2014 E sequence for either of the two N-linked glycosylation sites (residues 67 and 153) or for the pH dependent kl *β*-hairpin hinge region (residues 270 to 279) involved in viral fusion and activation of E protein dimer conformational change during cell entry^18^,^32^. In addition, Brun2014 reacted successfully with anti-DENV 1–4 complex HB114 and specific anti-DENV-1 monoclonal antibodies in IFA which targeted the E protein (4G2 and M17) or the non-structural protein 1 (HB114). This suggests retention of important antigenic epitopes and designation of Brun2014 within the DENV-1 serotype. Nonetheless, reduced reactivity of Brun2014 with neutralizing DENV-1 M17 monoclonal antibody was observed and this epitope interaction warrants further investigation.

Recently, traditional classification of the DENVs into four genetically distinct serotypes has been scrutinized and antigenic maps have inferred that these respective types are not antigenically homogeneous^33^. Whilst our IFA findings confer with the molecular characterization of Brun2014 as a DENV-1 virus, neutralization studies and broader investigations with DENV-1-4 immune sera will need to be conducted to further explore specific antigenic relatedness and immunological properties. Future studies should also examine how the observed amino acid changes impact on viral replication and pathological mechanisms and whether they contribute to altered vaccine and transmission control efficacies.

Examination of the Brun2014 3′UTR sequence revealed substantial nucleotide heterogeneity in the region immediately following the NS5 stop codon. This hypervariable region has been documented for several other DENV 1–4 strains and is a strong evolutionary marker^17^. The Brun2014 3′UTR hypervariable sequence was highly unconstrained and not reminiscent of the other DENV-1 3′UTR deletions previously reported^34^,^35^. This evidence is not surprising given the Brun2014 strain is evolutionarily distinct and diverged from the MRCA more than 300 years ago. Although further analysis of these findings and their biological significance is warranted, we hypothesize that there is little evolutionary pressure to conserve this region in contrast to the remaining 3′UTR sequence which is highly conserved in nature. Together with the high degree of sequence diversity observed for Brun2014 throughout the majority of the genome, these findings highlight that sylvatic DENV evolution continues to occur independently from that of human strains. Thus the risk of emergence of more pathogenic DENV viruses from sylvatic origins into the human population is entirely plausible^13^.

The importation of a pathogenic, transmissible and highly divergent DENV-1 genotype into Australia with a human population largely susceptible to DENV outbreaks could have significant public health implications. Fortunately, we identified the Brun2014 infection through the availability of accurate diagnostic capabilities such as our pan-DENV 1–4 TaqMan RT-PCR targeting the 3′UTR^36^. Indeed, with the exception of the 3′UTR hypervariable region, the 5′ and 3′UTR sequences of Brun2014 were extremely conserved compared to other DENV-1 strains indicating that PCR design in these regions could be highly appropriate for future detection of divergent sylvatic DENV strains.

Equally fortuitous was the fact that during the infectious period, the Brun2014 patient was residing in an area of Australia where *Ae. aegypti* does not occur and *Ae. notoscriptus* is the dominant container-inhabiting *Aedes* spp. This is critical, because Brun2014 can efficiently replicate in *Ae. albopictus* cells and disseminate in *Ae. aegypti* mosquitoes at rates not significantly different from the outbreak TSV08 strain. Importantly, acknowledgement should be given to the high variability existing between published DENV-1 infection rates in *Ae. aegypti* which range from 2.8 to 89%^37^,^38^. When infection rates are relatively low, outbreaks can still result when there are high numbers of the *Ae. aegypti* vector. This has been demonstrated with YFV in Africa^39^ and DENV-1 in North Queensland following the importation of the TSV08 outbreak strain, which resulted in 57cases (Jan Humphreys, personal communication). Indeed, if a sylvatic DENV strain was introduced by a viremic traveler, it is feasible that the *Ae. aegypti* strain in our study does have the capacity to facilitate an urban outbreak when mosquito population levels are high.

Previous studies suggest that sylvatic DENV emergence and spillover may be constrained by herd immunity among non-human reservoir hosts or the cross-protective immunity in humans following natural infection from endemic/epidemic DENV strains^5^. Nonetheless, despite these and similar serological studies using sylvatic DENV strains, recurring extinction of spillover strains could be overcome should sylvatic DENV amplification and dispersal occur at sufficient levels within an immunologically naïve population^5^,^11^. This could be further accelerated by substantial rises in ecotourism which accounts for ≈7% of global international travel and has led to more unprotected visitors returning from sylvatic DENV regions (Center for Responsible Travel, http://www.responsibletravel.org/news/fact_sheets/fact_sheet_-_global_ecotourism.pdf).

Other studies have also suggested that there is a low to non-existent adaptive barrier for emergence of sylvatic DENV in humans^11^,^21^. Although limited by available sylvatic sequences, we demonstrated similarly low selective pressures for respective DENV-1 sylvatic and endemic/epidemic datasets. We also compared relative replication rates of Brun2014 and TSV08 in mosquito, monkey and human cells in order to further investigate adaptation potential. However, these experiments are constrained by virus isolate and host cell parameters and do not fully explore infections following persistent mosquito and host cycle interactions. Several factors may also influence DENV replication rates during *in vitro* and *in vivo* experiments and significant differences among endemic/epidemic strains have been observed^40^,^41^. The finding that Brun2014 replicated at reduced rates in monkey or human cells compared to TSV08 is intriguing. This observation may indicate differences in host cell tropism which could have been influenced by the low passage, decreased cell adaptation or temperature sensitivity of Brun2014. Similar findings have been previously reported for replication comparisons of DENV-2 sylvatic and endemic/epidemic strains^42^. Of note, Vero cells are derived from the African monkey *Chlorocebus* sp. rather than the *Macaca* and *Presbytis* spp. implicated in Southeast Asian sylvatic DENV transmission^5^,^43^. However Vero cells and *in vitro* techniques are widely available and are useful for preliminary phenotypic experiments prior to investigations in non-human primates (NHPs). In the first study to investigate sylvatic DENV infection in NHPs, researchers have used a small number of African green monkeys to demonstrate that the viremia produced by infection with an African sylvatic DENV-2 strain was of similar magnitude and shorter in duration than the viremia produced by infection with a human endemic DENV-2 strain^44^. With the recent surge in human disease incidence and impending availability of a DENV vaccine, the ongoing study of sylvatic DENV transmission and replication provides a significant contribution to the understanding of DENV prevalence and pathogenicity. Clearly more investigations are required to further characterize sylvatic DENV infection dynamics and more accurately define the mechanisms leading to viremia and disease.

The Brun2014 patient was diagnosed with an acute illness and detection of flavivirus IgG antibodies in the day 5 serum suggested that this may have been a secondary DENV infection. Interestingly, the Brun2014 patient reported ongoing fever, photophobia, blurred vision and gait disturbance for up to six weeks following infection. Other severe manifestations including dengue hemorrhagic fever have been reported previously in association with sylvatic DENV infections^7^,^9^. The degree of risk and extent of disease severity pertaining to secondary sylvatic DENV infections following natural infection or post vaccination is currently unknown and therefore should not be discounted.

The emergence of sylvatic DENV strains also has the potential to influence disease mitigation strategies including vaccines and novel biological control strategies. Previous evidence with limited DENV sylvatic strains suggests that vaccination with endemic/epidemic DENV 1–4 strains may confer cross-protective immunity^11^,^43^. One very promising biological approach is the release of *Ae. aegypti* transinfected with the endosymbiotic bacterium *Wolbachia*. As *Wolbachia* inhibits replication of DENV, it is proposed that these releases could be used to suppress virus transmission in natural populations^45^,^46^. Given that *Wolbachia* not only inhibits DENV replication, but that of other RNA viruses, it is possible that the virus blocking phenotype would be expressed with sylvatic DENV strains as well.

Despite substantial advances in recent DENV research and development of control strategies, it is clear sylvatic DENV emergence remains an ongoing threat influenced by dynamic ecological, environmental and host behavioural patterns. This is highlighted by the isolation of the highly divergent Brun2014 strain which demonstrates that our knowledge of DENV evolution is not yet exhaustive and sylvatic DENV diversity has to date remained largely uncharacterized.

## Methods

### Ethics Statement

All methods were carried out in accordance with approved ethical guidelines.

Ethical approval for this study was granted by the FSS Human Ethics Committee. The patient has provided written informed consent for disclosure and dissemination of clinical and diagnostic laboratory findings.

### Case Description

A middle-aged female patient returned from Brunei to Brisbane, Australia, in 2014 and presented at a major hospital with fever, severe myalgias in the lower back and thighs, arthralgia, retro-orbital pain, photophobia and rash. Whilst in Brunei, the patient had been conducting research and over a two week period, was repeatedly exposed to mosquitoes during day and night expeditions from her urban base into the rainforest. Laboratory analysis conducted on an acute serum sample (day 5 post symptom onset) demonstrated the positive presence of flavivirus IgM and IgG antibodies, DENV positive NS1 antigen and DENV-1 positive RNA. Virus culture on the sample yielded a DENV-1 isolate which was further confirmed by IFA staining using specific DENV 1–4 monoclonal antibodies and nucleotide sequencing. A second convalescent serum collected 10 days following the first demonstrated positive cross-reactive DENV 1–4 IgM and flavivirus IgG antibodies.

### Virus culture

Acute phase patient serum was inoculated onto confluent monolayers of *Ae. albopictus* C6/36 cells (ATCC, CRL-1660) grown in Opti-MEM^^®^^ reduced serum growth medium (GM; Gibco BRL^^®^^, Invitrogen, California) supplemented with 0.2% bovine serum albumin. Cells were analysed for infection by IFA using both pan-flavivirus (4G2) and specific DENV 1–4 mouse monoclonal antibodies as previously described^47^. The panel of DENV monoclonal antibodies included DENV 1–4 complex (ATCC, HB114 D3-2H2-9-21), DENV-1 (ATCC, HB47 15F3-1), DENV-1 (M17^48^), DENV-2 (ATCC, HB46 3H5-1), DENV-3 (11D5^49^) and DENV-4 (ATCC, HB48 1H10-6). The recovered Brun2014 isolate was also cultured in mammalian Vero cells (ATCC, CCL-81). Concentrated, pelleted virus stocks of both Brun2014 (passage 3) and TSV08 (passage 5) were prepared in C6/36 cells as per previous methods^25^ and used for kinetic replication curve, whole genome sequencing and mosquito vector competence experiments. All cell lines used in the study tested negative for mycoplasma infection prior to experiments.

### Sequencing and phylogenetic analyses

Viral RNA extraction, RT-PCR and sequencing of DENV E genes and whole genomes were performed as previously described^25^,^50^,^51^. The 5′ and 3′ terminal ends of Brun2014 and TSV08 were amplified using the 5′/3′ RACE kit, 2^nd^ Generation Version 12 (Roche, U.S.A.) and sequenced using the Big Dye^®^ Terminator v3.1 cycle sequencing kit (Life Technologies, U.S.A) with primers specific for each virus (Supplementary Table S3) as per manufacturer’s instructions.

Sequence alignments were performed using ClustalW and MEGA 5.0 software^52^. To estimate the time to the MRCA, 100 DENV-1 E sequences from GenBank with known year and country of isolation were retrieved and analysed using a Bayesian Markov Chain Monte Carlo (MCMC) approach with BEAST package version 1.8.1^53^. The number of sequences studied afforded adequate Bayesian analysis as has been shown previously^9^,^10^,^15^. Highly homologous (>99%) or recombinant sequences (RDP v4.46 software, http://web.cbio.uct.ac.za/~darren/rdp.html) were excluded from the analysis to reduce data conflict and phylogenetic bias. The GTR + I + G nucleotide substitution model was chosen following scrutiny with JModelTest 2 software^54^ and the dataset was analysed using a relaxed uncorrelated lognormal molecular clock with a Bayesian skyline coalescent prior. The chains were run twice for 60,000,000 generations until convergence, sampling every 5,000 with 10% burn-in and combined using LogCombiner v1.8.1. Effective sampling size (ESS) values >200 were calculated and viewed with Tracer v1.6 (http://tree.bio.ed.ac.uk/software/tracer/). The tree data was summarised to create a target tree with TreeAnnotator version 1.8.1 and visualised in TreeGraph 2^55^. Similarly, 54 available DENV-1 coding sequences (10,179 nt) were analysed including Brun2014 and TSV08. For further classification of Brun2014 relative to other DENV serotypes, a maximum likelihood (ML) tree was also inferred from coding regions of 113 DENV sequences using MEGA 5.0 software^52^.

### Selection analysis

Assessment of selection pressures acting on the 3,392 aa of each of the 54 DENV-1 genome sequences was also performed using the Datamonkey web-server of the HyPhy package (www.datamonkey.org). Determination of ω ratios (*dN/dS*) was achieved using the following maximum likelihood methods namely: single-likelihood ancestor (SLAC), fixed effects likelihood (FEL), internal branch fixed-effects likelihood (IFEL) and random-effects method (REL)^56^. Codons were considered under positive selection where p values were ≤0.1 (SLAC, FEL or IFEL) or the Bayes factor was ≥50 (REL).

### Kinetic replication curve experiments

To ascertain relative replication curves for Brun2014 and TSV08 viruses, monolayers of C6/36, Vero and human hepatocyte cellular carcinoma (Huh-7) cells containing 4.2 × 10^5^ cells/well, 3.5 × 10^5^ cells/well and 4.5 × 10^5^ cells/well respectively were grown in 24-well microtiter plates and infected at a multiplicity of infection (m.o.i.) of 0.01. To calculate the m.o.i., viral titers of Brun2014 and TSV08 were determined by TCID_50_ assays using C6/36 cells.

The replication curve experiments were performed for each cell type by inoculating each virus onto 7 wells in triplicate followed by absorption for 1 hr at 28 °C (C6/36 cells) or 37 °C (Vero and Huh-7 cells). The culture fluid was removed and cell monolayers were washed with sterile PBS (pH 7.4) 3 times before addition of 500 μL/well of fresh medium supplemented with 3% foetal bovine serum (FBS). On each day between 1 and 7 days post infection, the culture supernatant was harvested from triplicate wells and frozen at −80 °C following removal of cells by centrifugation at 3,000 rpm for 5 min. Virus titers from each of the harvested culture supernatants were determined by performing TCID_50_ assays in 96 well microtiter plates seeded with confluent monolayers of C6/36 cells. The plates were incubated at 28 °C for 10 days, before they were fixed with PBS/acetone and stored at −20 °C. A fixed cell culture enzyme immunoassay (CCEIA) was used to detect DENV infection in the fixed C6/36 cells using the flavivirus-specific monoclonal antibody, 4G2^57^. Results of each of the triplicate samples are shown graphically as mean ± SE log_10_ TCID_50_/mL^58^ values taken at the respective time points. For each of the cell lines, the kinetic replication of Brun2014 and TSV08 was compared using a 2-way repeated-measures ANOVA on the triplicate titer values of each virus strain at each day post infection (GraphPad Software, Inc, San Diego, CA).

### Vector competence experiments

Female F_3_ *Ae. aegypti* from Cairns, Australia, and F_0_ *Ae. notoscriptus* from Brisbane, Australia, were exposed for 2 hours to infectious blood meals using a blood soaked pledget technique in three replicate experiments^59^. Each blood meal comprised stock virus diluted in commercially available defibrinated sheep blood and 1% sucrose to provide a final virus titer of approximately 10^7^ TCID_50_/mL.

To confirm the titer of virus at the time of feeding, 100 μL aliquots of the pre- and post-feeding blood/virus suspensions were diluted in 900 μL of GM +3% fetal bovine serum (FBS), containing antibiotics and antimycotics (Gibco BRL^^®^^, Invitrogen, California), before being stored at −80 °C. The pre- and post-feeding blood/virus mixtures were titrated as 10-fold dilutions in the wells of 96 well microtiter plates seeded with 75% confluent monolayers of C6/36 cells. The plates were incubated at 28 °C for 10 days, before they were fixed with PBS/acetone and stored at −20 °C.

Following feeding, blood engorged mosquitoes were placed in 900 mL gauze covered containers within an environmental growth cabinet. Mosquitoes were maintained at 28 °C, high humidity and 12:12 light:day, and provided 15% sucrose as a nutrient source. After 12 days, mosquitoes were killed with CO_2_ gas, and the legs and wings separately collected from the body. Recovery of virus from the legs and wings demonstrates a disseminated infection, whereby the virus has escaped from the midgut and has disseminated via the hemolymph through the hemocoel of the mosquito^60^. Disseminated infection rates are indicative of the vector competence of mosquitoes for DENV^28^,^29^. The bodies, and legs and wings were placed in separate 2 mL vials containing 1 mL of GM +3% FBS and a 5 mm stainless steel ball. The bodies, and legs and wings, were homogenized in a QIAGEN TissueLyser II (Qiagen, Hilden, Germany) prior to storing at −80 °C.

The mosquito homogenates were thawed, filtered through a 0.2 μm Supor^®^ membrane filter (Pall Corporation, Ann Arbor, MI) and inoculated in duplicate onto C6/36 cell monolayers in a 96 well microtiter plate. Plates were incubated and fixed as described above. All plates including those prepared for determining the initial titer of virus at the time of feeding were then analysed for DENV infection using the CCEIA and TCID_50_/mL calculations described above (kinetic replication curve experiments). Day 12 infection and dissemination rates for *Ae. aegypti* and *Ae. notoscriptus* exposed to the two DENV-1 strains were compared using a Chi-square test (GraphPad Software, Inc, San Diego, CA).

## Additional Information

**How to cite this article**: Pyke, A. T. *et al*. Highly divergent dengue virus type 1 genotype sets a new distance record. *Sci. Rep.* **6**, 22356; doi: 10.1038/srep22356 (2016).

## Supplementary Material

Supplementary Information

## Figures and Tables

**Table 1:** Infection and dissemination rates in *Ae. aegypti* and *Ae. notoscriptus* exposed to ≈10^7^ TCID_50_/mL of the DENV-1 Brun2014 and TSV08 strains, and tested at day 12 post-exposure.

Species	Infection^a^				Dissemination^b^			
	Brun2014		TSV08		Brun2014		TSV08	
*Aedes aegypti*	14.3	(8/56)	24.4	(11/45)	12.5	(7/56)	22.2	(10/45)
*Aedes notoscriptus*	9.4	(6/64)	23.9	(16/67)	1.6	(1/64)	6.0	(4/67)*

^a^Percentage of mosquitoes containing virus in their bodies (number positive/number tested).

^b^Percentage of mosquitoes containing virus in their legs and wings (number positive/number tested).

^*^Chi-square *P*-value < 0.05 for comparison between *Ae. aegypti* and *Ae. notoscriptus* for TSV08.
