# Supplementary material for: Highly divergent dengue virus type 1 genotype sets a new distance record
Source: Sci Rep. 2016 Feb 29;6:22356. doi: 10.1038/srep22356 (PMC4770315; doi:10.1038/srep22356)
Supplement: Supplementary Information [file srep22356-s1.pdf]

Supplementary Information

Manuscript Title: Highly divergent dengue virus type 1 genotype sets a new distance record

Author List: Alyssa T. Pyke[1*], Peter R. Moore[1], Carmel T. Taylor[1], Sonja Hall-Mendelin[1], Jane N. Cameron[1], Glen R. Hewitson[1], Dennis S. Pukallus[1], Bixing Huang[1], David Warrilow[1] and Andrew F. van den Hurk[1]

[1] Public Health Virology Laboratory, Forensic and Scientific Services, Coopers Plains, Queensland, Australia

Corresponding Author*: Alyssa.Pyke@health.qld.gov.au

Supplementary Table S1 Summary of DENV isolate sequences used in the study.

Serotype	Genotype	Year	GenBank accession number	Country	Strain
DENV-1	I	1943	AB074760	Japan	Mochizuki
DENV-1	I	1944	EU848545	Hawaii	VR-1254
DENV-1	II	1954	D10513	Thailand	TH-SMAN
DENV-1	II	1960	JF297570	Thailand	606147
DENV-1	II	1960	JQ922547	Thailand	DENV-1/THAI/606147/1960
DENV-1	V	1962	JF297572	India	62237
DENV-1	V	1963	JQ922544	India	IND/631288/1963
DENV-1	II	1963	AF425629	Thailand	2543-63
DENV-1	II	1964	AF180817	Thailand	16007
DENV-1	V	1968	AF425625	Nigeria	IBH 28328
DENV-1	V	1971	JQ922546	India	DENV-1/IND/715393/1971
DENV-1	V	1971	AY713473	Myanmar	D1.Myanmar.40553/71
DENV-1	III	1972	EF457905	Malaysia	P72-1244
DENV-1	IV	1974	U88535	Nauru	Nauru Island, Western Pacific
DENV-1	V	1976	AY722801	Myanmar	D1.Myanmar.40568/76
DENV-1	V	1977	JN379478	Grenada	CAREC 778164
DENV-1	V	1980	AY732476	Thailand	ThD1_0442_80
DENV-1	V	1980	AY732474	Thailand	ThD1_0673_80
DENV-1	I	1980	AF425630	Thailand	PUO 359
DENV-1	I	1981	AY732483	Thailand	ThD1_0008_81
DENV-1	IV	1983	AF425611	Australia	AUS HCS1
DENV-1	V	1985	GQ868601	British Virgin Islands	DENV-1/VG/BID-V2937/1985
DENV-1	V	1985	AF425620	Cote d'Ivoire	DAKAR A-1520
DENV-1	I	1987	AF425628	Taiwan	765101
DENV-1	IV	1988	AB600923	Indonesia	D1/JKTA88/88
DENV-1	V	1989	AF226687	French Guiana	FGA/89
DENV-1	I	1990	M87512	Singapore	Singapore S275/90
DENV-1	I	1990	JN638342	Thailand	D90-1197
DENV-1	V	1990	AF226685	Brazil	Den1BR/90
DENV-1	I	1991	AY732477	Thailand	ThD1_0336_91
DENV-1	V	1991	AF425626	Peru	DEI 0151
DENV-1	I	1991	AY732413	Thailand	ThD1_0119_91
DENV-1	IV	1991	FJ196845	China	GD03/91
DENV-1	V	1993	DQ285562	Comoros	Comoros 04.329/93
DENV-1	V	1993	AY153757	Costa Rica	cesara12
DENV-1	V	1993	AY762084	Singapore	Singapore 8114/93
DENV-1	I	1993	AY732461	Thailand	ThD1_K0022_93
DENV-1	I	1994	AB608789	Taiwan	360052
DENV-1	V	1994	AM746218	Saudi Arabia	945
DENV-1	I	1994	AY732480	Thailand	ThD1_0097_94
DENV-1	IV	1995	FJ196846	China	GD95/95
DENV-1	I	1995	JN638344	Thailand	00407/95
DENV-1	V	1995	AF425632	Venezuela	6222

DENV-1	V	1995	JN379486	Barbados	CAREC 9510153
DENV-1	IV	1995	DQ855297	China	GZ01/95
DENV-1	V	1996	AY722802	Myanmar	D1.Myanmar.23819/96
DENV-1	I	1997	JN638340	Thailand	30231/97
DENV-1	V	1997	AF311956	Brazil	BR/97-111
DENV-1	IV	1998	AB189121	Indonesia	98901530 DF DV-1
DENV-1	I	1998	AF309641	Cambodia	D1/H/IMTSSA/98/658
DENV-1	I	1998	AF298808	Djibouti	D1/H/IMTSSA/98/606
DENV-1	V	1998	GQ868559	Columbia	DENV-1/CO/BID-V3376/1998
DENV-1	I	1998	AY726555	Myanmar	D1.Myanmar.31459/98
DENV-1	IV	1998	AB189121	Indonesia	98901530 DF DV-1
DENV-1	V	1998	AF298807	Cote d'Ivoire	Abidjan
DENV-1	IV	1999	AY422785	Philippines	99SA236
DENV-1	V	2000	AF514883	Paraguay	259par00
DENV-1	V	2000	AF514889	Argentina	297arg00
DENV-1	I	2000	AY620951	Myanmar	My00D136393
DENV-1	IV	2000	JN415515	Palau	Palau 2000
DENV-1	IV	2000	JN415499	East Timor	ET00 243
DENV-1	IV	2001	DQ672564	Hawaii	HawO3663
DENV-1	I	2001	AY713476	Myanmar	D1.Myanmar.305/01
DENV-1	I	2001	AY732479	Thailand	ThD1_0102_01
DENV-1	I	2001	AY732464	Thailand	ThD1_K0407_01
DENV-1	IV	2001	AY630407	French Polynesia	FP/01/192206
DENV-1	IV	2001	DQ672564	Hawaii	HawO3663
DENV-1	IV	2002	KF559254	Myanmar	M49440
DENV-1	IV	2002	JN415503	Fiji	Fiji 2002
DENV-1	IV	2002	EU863650	Chile: Easter Island	CHI3336-02
DENV-1	IV	2003	FJ196842	China	GD66/03
DENV-1	V	2003	JN379472	Barbados	CAREC 0308216
DENV-1	IV	2003	JN415488	Indonesia	Bali 2003
DENV-1	IV	2003	AB195673	Seychelles	NIID03-41
DENV-1	V	2004	EU596501	Nicaragua	DENV-1/NI/BID-V653/2004
DENV-1	V	2004	JN415524	Sri Lanka	Sri Lanka 2004
DENV-1	I	2004	FR666922	Malaysia	D1/Malaysia/33087/04
DENV-1	I	2004	AM746216	Saudi Arabia	6633
DENV-1	I	2004	AY835999	China	ZJ01/2004
DENV-1	IV	2004	AY858983	Indonesia	SC01
DENV-1	IV	2004	AB204803	Federated States of Micronesia (Yap)	NIID04-27
DENV-1	V	2005	EU081258	Singapore	D1/SG/05K4147DK1/2005
DENV-1	I	2005	EU081226	Singapore	D1/SG/05K814DK1/2005
DENV-1	III	2005	FN825674	Malaysia	D1/Malaysia/36046/05
DENV-1	V	2005	EU179860	Brunei	DS06-210505
DENV-1	V	2005	JF297581	India	55290
DENV-1	IV	2005	JN415516	Philippines	Philippines 2005
DENV-1	V	2005	JQ922548	India	DENV-1/IND/55290/2005
DENV-1	I	2006	FJ196844	China	GD02/06

DENV-1	V	2006	HQ332182	Venezuela	VE_61006_2006
DENV-1	I	2006	JN415533	Vietnam	Vietnam 2006
DENV-1	I	2007	JN415523	South East Asia	Southeast Asia 2007
DENV-1	V	2007	GU131962	Mexico	DENV-1/MX/BID-V3669/2007
DENV-1	I	2008	KC172829	Laos	XB998_Laos_2008
DENV-1	I	2008	AB608787	Taiwan	SDDF1543
DENV-1	V	2008	JN415506	Guyana	Guyana 2008
DENV-1	V	2008	GU131863	Brazil	DENV-1/BR/BID-V3490/2008
DENV-1	V	2008	JN903579	India	D1/IN/RGCB419/2008
DENV-1	V	2008	GQ357692	Singapore	SG(EHI)DED65008
DENV-1	I	2008	GU131792	Vietnam South	DENV-1/VN/BID-V4034/2008
DENV-1	I	2008	JN415521	Singapore	Singapore 2008
DENV-1	I	2008	JN415527	Thailand	Thailand 2008b
DENV-1	I	2008	JN415534	Vietnam	Vietnam 2008a
DENV-1	I	2008	KR919821	Australia	TSV08
DENV-1	V	2009	KC692495	Argentina	HNRG12188
DENV-1	V	2009	JN903581	India	D1/IN/RGCB592/2009
DENV-1	V	2009	JF960211	Singapore	SG(EHI)D1/0091Y09
DENV-1	I	2009	JN415519	PNG	PNG 2009
DENV-1	I	2009	GU131895	Cambodia	DENV-1/IPC/BID-V3787/2009
DENV-1	I	2009	HQ891316	Sri Lanka	DV1_SL_2009d
DENV-1	IV	2010	JQ915080	New Caledonia	NC10/080810-1138
DENV-1	I	2010	HG316481	Thailand	KDH0026A
DENV-1	I	2010	HG316482	Thailand	KDH0030A
DENV-1	V	2010	KC692512	Argentina	HNRG25001
DENV-1	I	2010	KC182084	Laos	LNT1975_Laos_2010
DENV-1	IV	2010	KR919811	Philippines	Phil 2010
DENV-1	IV	2010	JN415492	Indonesia	Bali 2010d
DENV-1	IV	2010	JN415494	Indonesia	Bali 2010f
DENV-1	IV	2010	JN415513	Malaysia	Malaysia 2010
DENV-1	V	2010	KJ189367	Puerto Rico	DENV-1/PR/BID-v8188/2010
DENV-1	V	2010	JQ675358	USA	DENV-1/BOL-KW010
DENV-1	I	2011	KJ649286	Saudi Arabia	DENV-1-Jeddah
DENV-1	I	2011	KR919805	Indonesia	Bali 2011
DENV-1	I	2011	KC848576	Somalia	SO/DB118/2011
DENV-1	IV	2011	KR919815	Papua New Guinea	PNG 2011
DENV-1	IV	2011	JX298570	Fiji	Fiji 2011b
DENV-1	V	2011	KF289072	India	RR107
DENV-1	IV	2012	KJ933413	China	Su1
DENV-1	I	2012	KJ726662	Sri Lanka	SL_2012_GS0319
DENV-1	I	2012	KR919808	Australia	Cairns 2012
DENV-1	IV	2012	KR919819	Philippines	Phil 2012
DENV-1	IV	2012	KR919813	Indonesia	Indo 2012
DENV-1	V	2012	KF973455	Nicaragua	DENV-1/NI/BID-V7650/2012
DENV-1	V	2013	KF864667	China	Zj/yw01
DENV-1	I	2013	KR919806	Indonesia	Bali 2013

DENV-1	I	2013	KR919807	Thailand	Thai 2013
DENV-1	I	2013	KR919809	Cambodia	Cam 2013
DENV-1	I	2013	KR919817	Vanuatu	Van 2013
DENV-1	I	2013	KF184975	Angola	Angola_2013
DENV-1	V	2014	KM458188	USA	US/DB167/2014
DENV-1	I	2014	LC002828	Japan	D1/Hu/Saitama/NIID100/2014
DENV-1	I	2014	KR919816	East Timor	ET2014
DENV-1	IV	2014	KR919812	Indonesia	Bali 2014
DENV-1	IV	2014	KR919818	Papua New Guinea	PNG 2014b
DENV-1	IV	2014	KR919814	Papua New Guinea	PNG 2014a
DENV-1	VI - Sylvatic	2014	KR919820	Brunei	Brun2014
DENV-1	IV	2015	KR919810	Australia	Tully 2015
DENV-2	Asian II	1944	AF038403	Papua New Guinea	New Guinea C
DENV-2	Asian I	1964	U87411	Thailand	16681
DENV-2	Sylvatic	1966	EF105387	Nigeria	IBH11208
DENV-2	Sylvatic	1966	EU003591	Nigeria	IBH11234
DENV-2	Sylvatic	1966	EF105388	Nigeria	IBH11664
DENV-2	Sylvatic	1970	EF105384	Senegal	Dak HD 10674
DENV-2	Sylvatic	1970	EF105379	Malaysia	P8-1407
DENV-2	Asian I	1974	DQ181806	Thailand	ThD2_0038_74
DENV-2	Sylvatic	1974	EF105385	Senegal	Dak Ar D20761
DENV-2	American	1977	EU056812	Puerto Rico	1328
DENV-2	Sylvatic	1980	EF105382	Burkina Faso	Dak Ar 2039
DENV-2	Sylvatic	1980	EF105380	Cote d'Ivoire	Dak Ar 578
DENV-2	Sylvatic	1980	EF105383	Cote d'Ivoire	Dak Ar A1247
DENV-2	Sylvatic	1980	EF105386	Burkina Faso	Dak Ar A2022
DENV-2	Sylvatic	1980	EF105381	Cote d'Ivoire	Dak Ar 510
DENV-2	Sylvatic	1981	EF105378	Guinea	PM33974
DENV-2	Asian/American	1983	M20558	Jamaica	Jamaica/N.1409
DENV-2	Cosmopolitan	1993	AY037116	Australia	TSV01
DENV-2	American	1994	JX966379	Mexico	DENV2-QR94
DENV-2	Asian I	1998	DQ181799	Thailand	ThD2_0017_98
DENV-2	Asian/American	1998	AF208496	Martinique	DEN2/H/IMTSSA-MART/98-703
DENV-2	Cosmopolitan	1999	AF359579	China	FJ11/99
DENV-2	Sylvatic	1999	EF105389	Senegal	Dak Ar 141069
DENV-2	Sylvatic	1999	EF105390	Senegal	Dak Ar 141070
DENV-2	Sylvatic	1999	EF457904	Senegal	Dak Ar D75505
DENV-2	Cosmopolitan	2005	EU179858	Brunei	DS04-221205
DENV-2	Sylvatic	2008	FJ467493	Malaysia	DKD811
DENV-2	Sylvatic	2009	JF260983	Guinea-Bassau	EEB-17
DENV-2	Asian/American	2011	KC294207	Peru	DENV-2/PE/FPI01202/2011
DENV-3	V	1956	M93130	Philippines	H87
DENV-3	V	1980	AF317645	China	80-2
DENV-3	II	1987	AY676353	Thailand	ThD3_0007_87
DENV-3	III	1989	JQ411814	Sri Lanka	UNC3001
DENV-3	I	1994	AY744685	French Polynesia	PF94/136116

DENV-3	II	1994	AY923865	Thailand	C0360/94
DENV-3	I	1995	DQ675519	Taiwan	95TW466
DENV-3	II	1998	JN406514	Australia	Cairns 98
DENV-3	III	1999	AY099337	Martinique	D3/H/IMTSSA-MART/1999/1243
DENV-3	III	2000	AY099336	Sri Lanka	D3/H/IMTSSA-SRI/2000/1266
DENV-3	III	2004	EF629367	Brazil	BR DEN3 97-04
DENV-3	III	2005	EU081198	Singapore	D3/SG/05K2933DK1/2005
DENV-3	III	2006	HQ332171	Venezuela	VE_61035_2006
DENV-3	I	2008	JN406515	Australia	Cairns 2008
DENV-3	II	2010	HG316484	Thailand	KDH0014A
DENV-4	I	1961	JF262783	India	INDIA G11337
DENV-4	Sylvatic	1973	JF262780	Malaysia	P73-1120
DENV-4	Sylvatic	1975	EF457906	Malaysia	P75-215
DENV-4	Sylvatic	1975	JF262779	Malaysia	P75-514
DENV-4	II	1981	AF326573	Dominica	Dominica 1981
DENV-4	II	1994	JF262782	Haiti	Haiti73
DENV-4	II	1995	JF262781	Venezuela	INH6412
DENV-4	III	1997	AY618989	Thailand	ThD4_0017_97
DENV-4	II	2000	AY618993	Thailand	ThD4_0734_00
DENV-4	I	2001	AY618992	Thailand	ThD4_0485_01
DENV-4	I	2009	KF041260	Pakistan	D4/Pakistan/150/2009
DENV-4	II	2010	JQ822247	China	GZ30
DENV-4	II	2010	JN983813	Brazil	Br246RR/10
DENV-4	II	2010	JX024758	Singapore	EHI310A129SY10

Supplementary Table S2. Summary of significant amino acid substitutions between Brun2014 and a consensus DENV-1 sequence[a].

Gene	Site[b]	Amino acid substitution[c]	Unique amino acid substitutions[d]
Capsid	39	S → T	*
	99	K → R	HQ891316<br>KJ726662
	101	S → T	EF457905
	103	T → V	*
	104	M → A	*
	111	T → M	*
	113	L → F	*
	114	A → S	*
prM	15	S → N	EF457905
	29	A → G	*
	31	V → I	AF514889
	43	E → D	*
	79	T → I	*
	82	Q → P	*
	89	D → E	KF864667<br>KC692512<br>KJ189367
M	16	T → A	AB074760<br>EU848545<br>JQ922547<br>EF457905
	52	A → I	*
	62	I → V	*
E	6	I → V	EF457905<br>FN825674
	51	T → S	AY732461
	64	K → R	DQ672564
	81	T → S	*
	83	V → S	*
	120	K → E	EF457905<br>FN825674
	122	V → L	*
	125	L → M	*
	157	E → G	EF457905<br>FN825674
	221	T → A	*
	226	T → S	*
	227	S → P	AB074760<br>EU848545
	309	E → K	AB074760
	325	K → E	*
	338	S → T	*
	378	I → M	*

	382	A → V	JN415527<br>JN415503
	384	E → D	*
	398	I → L	*
	425	I → V	GU131792<br>AY835999
	461	I → L	*
	484	M → I	*
	485	V → I	AB195673
NS1	2	S → T	JN903579
	6	I → V	AB074760
	52	E → D	*
	72	S → A	*
	77	H → Y	GQ868601
	94	N → K	*
	103	M → G	*
	146	D → S	EF457905
	192	K → R	AB074760
	213	A → S	*
	290	H → Q	*
NS2a	4	E → K	*
	7	S → N	KF864667<br>KF184975
	19	I → V	KF864667<br>KF184975
	29	R → G	*
	37	L → M	*
	44	I → V	GU131962
	56	L → V	AY722802
	63	N → H	*
	65	S → L	*
	67	R → S	*
	83	M → I	*
	87	F → L	*
	95	R → K	JQ922547
	101	V → I	*
	111	V → I	*
	112	A → T	*
	113	S → P	*
	120	L → I	*
	143	H → Y	*
	168	M → A	*
	194	V → I	*
	210	T → S	*
	218	K → R	GU131895<br>AB189121
NS2b	4	L → I	HQ332182<br>GQ868601<br>KF973455

	18	L → M	*
	39	I → V	JQ922547
	52	S → T	*
	56	A → T	*
	71	S → A	*
	92	D → E	*
	102	T → A	*
	106	V → L	*
NS3	7	T → I	*
	30	L → V	*
	31	L → F	*
	59	M → T	*
	91	T → V	*
	112	A → V	*
	119	P → H	*
	143	E → D	*
	146	I → V	*
	168	A → S	*
	170	A → V	*
	187	R → K	*
	212	A → S	*
	216	K → R	*
	239	M → L	*
	249	K → R	*
	250	S → N	*
	323	V → A	*
	331	A → S	*
	334	Q → Y	*
	338	R → K	EU863650<br>AB195673
	356	P → Q	*
	368	S → T	*
	401	N → S	*
	405	Y → F	*
	436	K → R	*
	467	N → S	*
	556	F → I	*
	559	S → A	*
	562	R → K	EU848545
	573	V → I	*
NS4a	15	Q → H	*
	60	L → M	*
	63	V → T	*
	69	T → A	*
	110	I → V	*
2K	16	F → V	*
	17	M → L	*
NS4b	17	H → S	*

	22	N → I	*
	23	H → Q	AF514889
	26	A → T	*
	28	M → I	AF311956
	53	M → L	EF457905
	64	I → M	*
	106	L → M	*
	133	E → D	*
	163	A → T	KF184975<br>KF864667<br>JQ675358
	191	L → F	*
	247	G → N	*
NS5	23	S → L	*
	39	S → T	*
	52	K → R	GU131792
	181	I → V	DQ672564
	195	Q → R	EF457905<br>KJ189367
	245	A → T	EF457905<br>AF514889<br>KF289072
	253	R → K	AY726555
	372	I → V	*
	375	V → I	*
	378	K → G	*
	382	G → K	*
	387	N → K	AY722802
	426	E → D	*
	435	H → N	*
	500	E → D	*
	522	S → A	*
	523	K → R	JQ922547
	544	E → D	*
	551	K → R	GU131895
	552	I → V	EF457905
	554	D → E	GU131962
	555	I → L	*
	559	E → D	*
	562	L → Q	AY835999
	569	K → R	*
	583	A → G	*
	627	I → V	*
	640	E → N	*
	697	K → R	*
	830	S → T	*
	853	T → S	*
	860	T → I	*
	871	R → K	*

877	N → H	*
879	L → Q	*
886	K → R	*
893	D → E	AY835999<br>HQ891316<br>KJ726662

[a]Amino acid comparison (3392 residues) of Brun2014 with a consensus sequence derived from DENV-1 sequences representing each of the five DENV-1 genotypes. [b]Amino acid numbering is given from the start of each gene.

[c]Amino acid substitutions are shown with residues from the DENV-1 consensus sequence on the left and those from Brun2014 on the right.

[d]Asterisk (*) denotes amino acid substitutions which are unique to Brun2014. GenBank accession numbers are also given for DENV-1 strains which contained the same amino acid substitutions as Brun2014.

Supplementary Table S3 5’/3’ RACE primers used in the study.

	Primer name	Sequence[a]
5’ RACE	Bru 576r	5’-^{576}TCTTCACATAGATCCCCCAAGTCC^{553}-3’
	Bru 540r	5’-^{540}GTGCACATGTTAATACCCCCT-^{520}3’
	Towns 480r	5’-^{480}CTGACTATCATGTGCGGCTCTC^{459}-3’
	Towns 451r	5’-^{451}TGTAGTCAAATGGAACGCCAAG^{430}-3’
3’ RACE	Bru 10427f	5’-^{10,427}GGCTTGAGCAAACCGTGCT^{10,445}-3’
	Bru 10474f	5’-^{10,474}AACCTGGGAGGCTGCAAT^{10,491}-3’
	Towns 10248f	5’-^{10,248}TCAAGAACGAGAGTGATCCCG^{10,268}-3’
	Towns 10325f	5’-^{10,325}AACAAGGCAAGAAGTCAGGCC^{10,345}-3’

[a]Primer sequences are given with corresponding genome nucleotide positions.

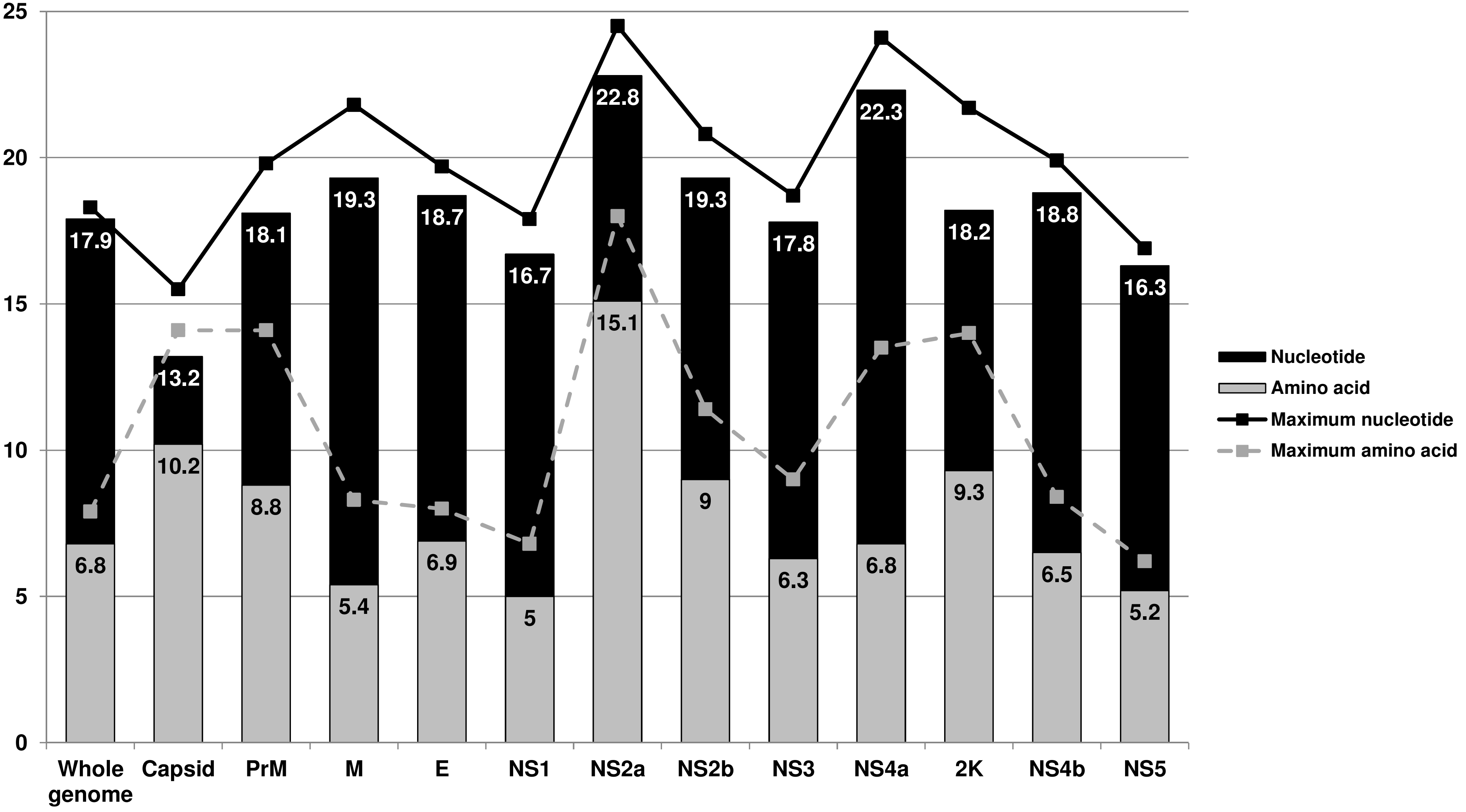
25
20
15
10
5
0
17.9
6.8
13.2
10.2
18.1
8.8
19.3
5.4
18.7
6.9
16.7
5
22.8
15.1
19.3
9
17.8
6.3
22.3
6.8
18.2
9.3
18.8
6.5
16.3
5.2
Whole genome
Capsid
PrM
M
E
NS1
NS2a
NS2b
NS3
NS4a
2K
NS4b
NS5
Nucleotide
Amino acid
Maximum nucleotide
Maximum amino acid

Supplementary Figure S1. Sequence diversity between Brun2014 and 53 dengue virus serotype 1 (DENV-1) strains. Average pairwise differences for the DENV-1 whole genome coding region and individual viral proteins are shown numerically for both nucleotide and amino acid sequence comparisons. Maximum pairwise differences are also plotted for nucleotide (solid line) and amino acid (dashed line) sequences respectively. Among individual viral proteins, nonstructural protein (NS) 2a was found to contain the highest nucleotide (22.8%) and amino acid (15.1%) differences. Alternatively, the least nucleotide and amino acid differences were demonstrated in the capsid (13.2%) and NS1 (5.0%) proteins respectively. Of note, estimated differences for the E protein were 18.7% (maximum 19.7%) for nucleotide and 6.9% (maximum 8.0%) for amino acid which were almost identical to the nucleotide (18.6%, maximum 19.9%) and amino acid (7.0%, maximum 8.2%) differences obtained with 100 DENV-1 sequences.

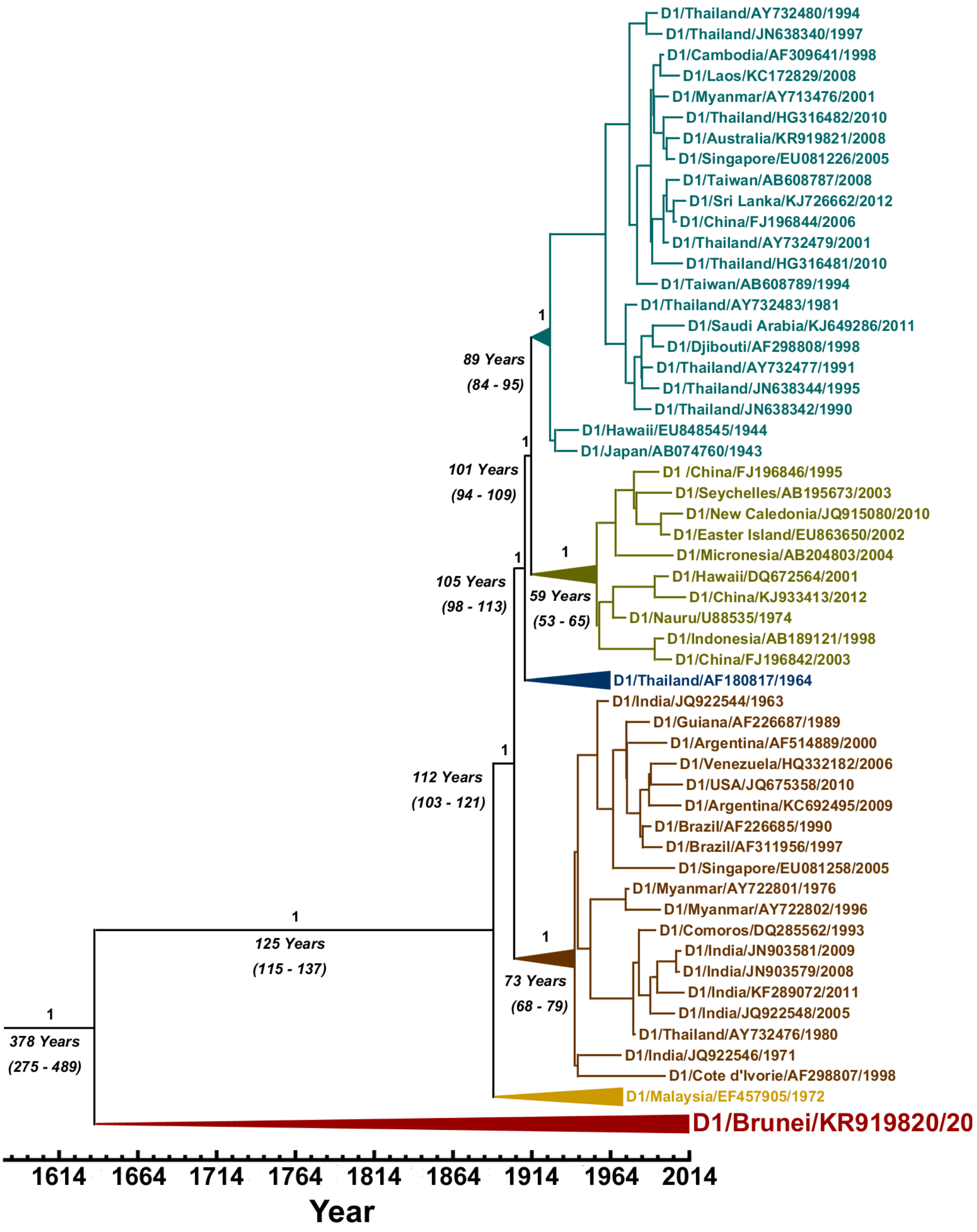

Supplementary Figure S2. Maximum clade credibility tree of 54 DENV-1 whole genome coding region sequences. All five major DENV-1 genotypes (I to V) are shown and compared to a new postulated sylvatic genotype (VI) containing the previously unknown strain, Brun2014. Horizontal branch lengths are drawn to scale and are proportional to time. Posterior probability values (1.0) are shown for key nodes together with corresponding divergence times and respective 95% HPD values (in parentheses).
